# A Comprehensive Review: Materials for the Fabrication of Optical Fiber Refractometers Based on Lossy Mode Resonance

**DOI:** 10.3390/s20071972

**Published:** 2020-04-01

**Authors:** Aritz Ozcariz, Carlos Ruiz-Zamarreño, Francisco J. Arregui

**Affiliations:** 1 Department of Electric, Electronic and Communication Engineering, Public University of Navarre, E-31006 Pamplona, Spain; 2 Institute of Smart Cities (ISC), Public University of Navarre, E-31006 Pamplona, Spain

**Keywords:** optical fiber sensor, optical sensor, LMR, lossy mode resonance

## Abstract

Lossy mode resonance based sensors have been extensively studied in recent years. The versatility of the lossy mode resonance phenomenon has led to the development of sensors based on different configurations that make use of a wide range of materials. The coating material is one of the key elements in the performance of a refractometer. This review paper intends to provide a global view of the wide range of coating materials available for the development of lossy mode resonance based refractometers.

## 1. Introduction

Sensors have acquired an unprecedented presence in modern society. In industry, medicine, and even in our own homes, we are increasingly relying on a number of sensors to achieve a safer environment and a healthier and more comfortable life [1,2,3,4]. As this field evolves, new mechanisms and configurations are researched, which facilitate the development of better sensors and new applications.

The versatility and practicality of optical fibers makes them a great base for the fabrication of sensors. In fact, there is a wide range of mechanisms that allow the fabrication of sensors using optical fibers. This review paper will discuss one type that has already proven to be capable of achieve astonishing performance—lossy mode resonance (LMR) fibers. LMR occurs due to the interaction of the light propagating through a waveguide coated with a film of appropriate optical properties. If the coating possesses certain properties (detailed below), the waveguide modes may couple to the coating modes, generating a lossy mode. This effect causes an absorption peak at the wavelength at which the conditions are fulfilled. This resonance should not be confused with surface plasmon resonance (SPR), which can also be observed in waveguides with certain coatings, since they are two separate phenomena. SPR is generated at metal–dielectric interfaces, which are typically obtained with conductive thin films, such as gold [5], whereas the LMR conditions enable the use of a larger range of materials [6]. LMR can be supported by the fabrication of films made of different materials, such as indium tin oxide (ITO) [7], zinc oxide [8], polymers [9], or silicon nitride [10], among others.

LMR will suffer a variation if either a variation in the properties of the LMR support film (either refractive index or thickness) or a change in the optical properties (refractive index) of the surrounding media occurs. Provided the coating is stable, the LMR will only be affected by the surrounding media refractive index (SRI), meaning that it is essentially a good mechanism for the development of refractometers. This mechanism can be used as the basis for the design of multiple kinds of physical and chemical sensors. An external coating, whose optical properties rely on a given parameter that needs to be measured, could be fabricated on top of a LMR-based refractometer, and the variation of this variable could be measured by monitoring the LMR wavelength shift. It should be noted that the development of such sensors does not rely solely on the fabrication of a functionalizing overlay. Given the number of materials capable of supporting LMR, the right choice for the composition of this coating can facilitate the fabrication of many different kinds of sensors. For instance, an ITO nanoparticle coating was used for the fabrication of a hydrogen gas sensor [7], while zinc oxide nanorods were used in the fabrication of a coating whose LMR was sensitive to sulfide gas (H_2_S) [11]. On a different note, polymeric coatings of polyallylamine hydrochloride (PAH) and polyacrylic acid (PAA) were used to fabricate LMR-based pH [12] and relative humidity [9] sensors.

The purpose of this review is to summarize the most relevant coating materials that have been reported for the development of LMR-based sensors. We also aim to provide an overview of the results achieved with each of them and the applications proposed for each material. The study of the different coating materials presented here is a proof of the versatility and great potential of this technique, as it has a great range of applications.

## 2. Basic Concepts

**Figure 1:**
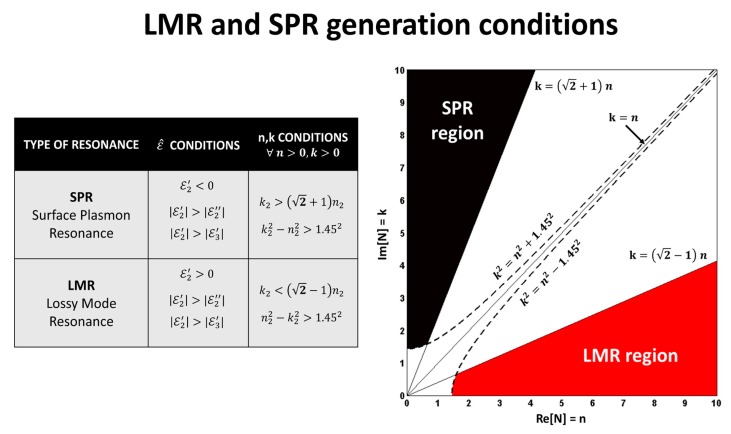
Optical parameters for the generation of surface plasmon resonance (SPR) and lossy mode resonance (LMR). Reprinted from [13] with permission from Elsevier.

A classical optical fiber is typically composed of a cylindrical core surrounded by a layer known as cladding. The lower refractive index of the cladding with respect to the core allows the light to propagate through the fiber with very low attenuation and in a way that is practically insensitive to the external conditions of the fiber, making it the ideal mechanism for transmission of optical signals over long distances. If the cladding (or a part of it) is removed in a segment of the fiber and substituted for a different material, the propagation of the light might be affected. Depending on the optical properties of the applied coating, different resonance might be excited. One of the most well-known resonances generated in this way is surface plasmon resonance (SPR), which occurs when the real part of the coating permittivity is negative and greater in magnitude than both its imaginary part and the real part of the external medium permittivity [13]. A conductor such as gold is the typical material used for the fabrication of SPR-supporting thin films. Another type of resonance that can be generated with materials of different optical properties is LMR. In order to be able to generate LMR, the coating must have a real positive permittivity part that must also be greater in magnitude than both its imaginary part and the real part of the external medium permittivity. In terms of the refractive index, this means that the chosen coating must have a relatively high real part (n) and a comparatively low (but non zero) imaginary part (k) (Figure 1).

**Figure 2:**
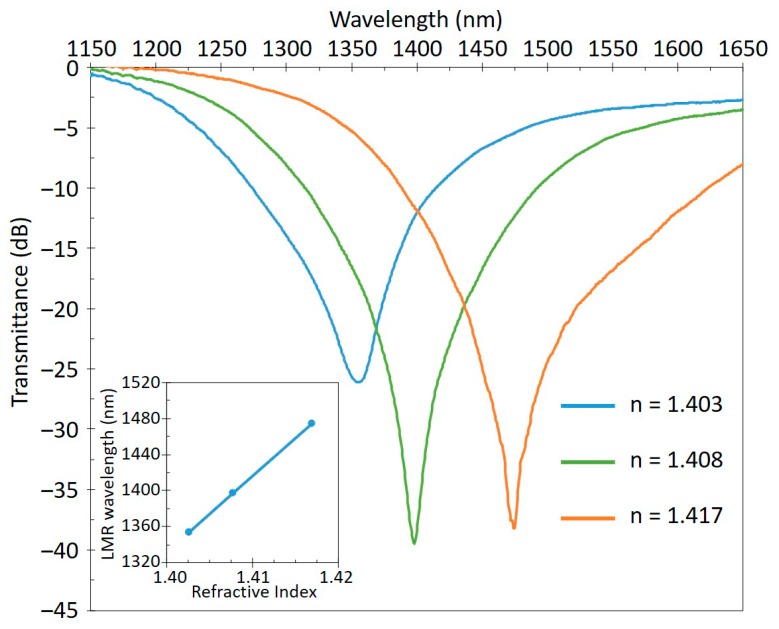
Example of the evolution of LMR for different surrounding media refractive index (SRI) values. The peak of the LMR shifts to longer wavelengths as the SRI increases. The inset shows a typical calibration curve, plotting the LMR position in function of the SRI, which characterizes the device as a refractometer.

When LMR is generated in an optical fiber, this phenomenon can be observed as an absorption peak at a given wavelength in the transmission spectra. The location of this resonance in the spectra depends on the optical properties (refractive index) of the LMR support film, its thickness, and the surrounding media refractive index (SRI). Therefore, a variation in the SRI or in the film (either its thickness or its refractive index) will induce a shift in the wavelength of the LMR that can be measured, and is the basis for the fabrication of a number of sensors. Figure 2 shows an example of the spectra of a LMR sensor measuring different SRI values. The peak of the LMR shifts to a longer wavelength as the SRI increases and its position can be tracked, establishing a direct relation between LMR position and SRI value (inset in Figure 2). It is important to note that the sensitivity to SRI (measured as the wavelength shift in nanometers per refractive index unit, nm/RIU) is not a constant, since it increases for higher SRI values [6]. Additionally, it can be observed that an increase in the coating thickness causes the shift on the LMR to a longer wavelength, which also provokes an increase in sensitivity. It should also be noted that as the thickness increases, more LMR of higher orders appear at shorter wavelengths, and these new resonances are consecutively less sensitive than the previous one. Another factor to consider regarding the sensitivity of the LMR is the refractive index of the coating. A coating with a higher refractive index will generate a more sensitive resonance. It can be easily inferred that the appropriate choice and fabrication of this coating material will have a direct impact on the performance of the sensor.

### Setup Configurations

**Figure 3:**
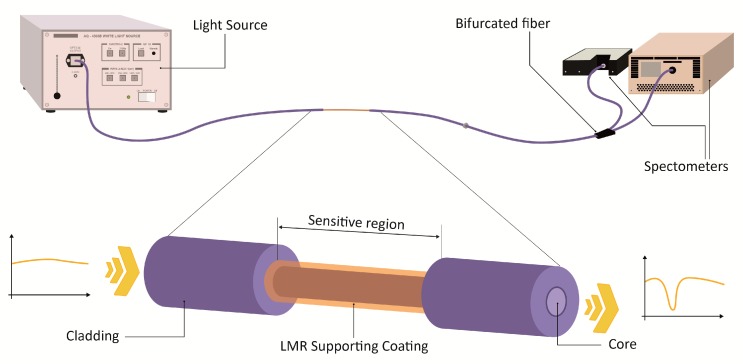
Typical setup involving a cladding-removed multimode optical fiber (CRMOF) for the fabrication of a LMR-based refractometer. In this case, a bifurcated fiber was included to observe a wider spectrum using two refractometers.

A relevant aspect to take into consideration is the architecture or setup designed for the sensor. It is important to note that LMR, unlike SPR, occurs in both transverse electric (TE) and transverse magnetic (TM) modes. These modes refer to the directions of the electric and magnetic fields in the waveguide. A TE mode has no electric field in the direction of propagation (magnetic only), while the TM mode has no magnetic field in the direction of propagation (electric field only). In order to excite a surface plasmon, the incident wave must have an electric field component parallel to the direction of propagation of the wave at the waveguide–coating interface, which is the case for the TM mode only [5]. In this way, the study of SPR requires the use of polarized light so that only the TM mode is propagated, which allows the full amplitude of the resonance to be observed. On the contrary, LMR is based on the coupling of a guided mode to a mode with losses on film, and is not limited to a single mode of propagation [6]. This provides greater flexibility in the experimental setups, as no polarization of light is needed. Therefore, a basic probe configuration for LMR sensor fabrication consists of a segment of cladding-removed multimode optical fiber (CRMOF), on top of which a high refractive index thin film is deposited [6]. The coated CRMOF is then connected to a light source and an optical spectrum analyzer (OSA) or a spectrometer in order to study the transmitted spectra (Figure 3). A similar approach using a single mode fiber (SMF) would require an etching process be applied to the fiber in order to reduce the diameter of the cladding so that the evanescent mode in the fiber could reach the later deposited LMR support coating. This process is necessary in standard SMFs because the small diameter of the core in such fibers makes it very difficult to complete the removal of the cladding, in contrast to multimode fibers (MMFs), which can be specifically fabricated so that the cladding can easily be completely removed [6,10,11]. Alternatively, an optical fiber could be tapered in order to reduce its diameter so that the light is guided out of the core and can interact with an outer coating. This configuration has been used in the design of reflection setups, where a tapered tip coated with a LMR support material is suggested [14,15].

**Figure 4:**
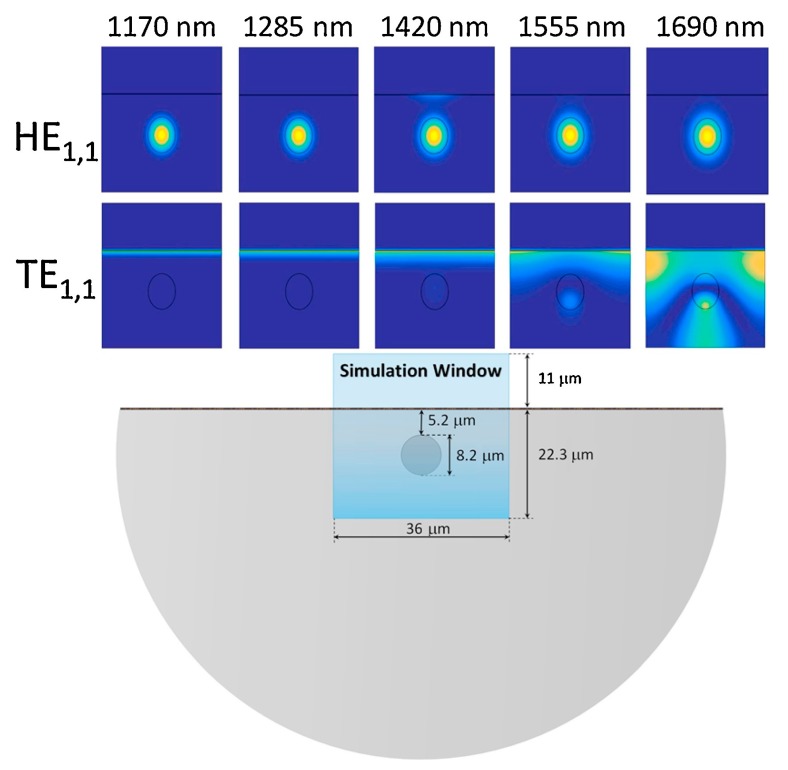
Schematic and simulation results of the electric field intensity in an indium tin oxide (ITO)-coated D-shaped fiber for the TE_1,1_ and HE_1,1_ modes. Reprinted from [16] with permission from Elsevier.

A more complex setup for the purpose of separating the propagation modes involves the use of polarized light and a D-shaped fiber. This kind of fiber contains a segment where a polishing process has been performed. This procedure modifies the geometry of the fiber, whose section acquires a capital “D” shape (hence its name). This configuration, in combination with the use of polarized light, causes the LMR to be excited only at a certain axis (the axis with the polished side), as it is the only angle where the wave reaches the outer coating. This mechanism allows the effective separation of LMR generated in different propagation modes. As an example, Figure 4 presents the simulation of two propagation modes in an ITO-coated D-shaped fiber [16]. It shows how the cladding mode TE_1,1_ (a particular TE mode observed on circular waveguides) is confined in the cladding at a wavelength of 1690 nm, and transforms into a TE mode on the ITO layer as the wavelength decreases. It also shows how the HE_1,1_ (a hybrid mode where the TE component dominates over the TM) is confined in the core for all the simulated wavelengths, with the exception at 1420 nm, where it can be seen how part of the energy couples to the ITO coating due to the LMR.

**Figure 5:**
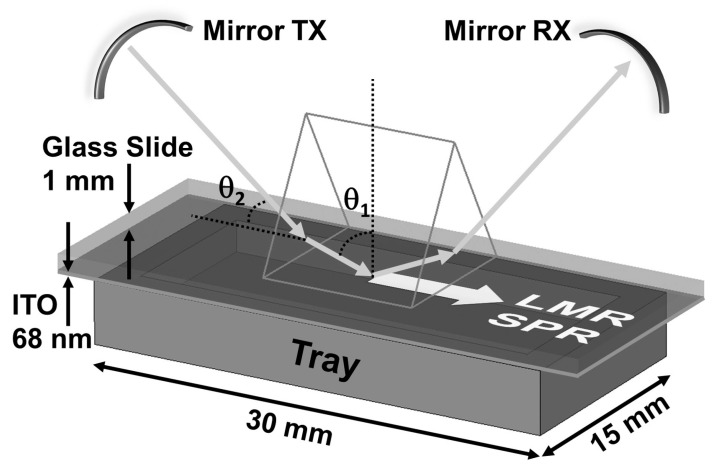
Example of a Kretschmann configuration for the analysis of SPR and LMR. In this case, the polarized light coming from the transmitting mirror TX is coupled through a prism to a coated glass slide so that the light beam is reflected in the interface at the desired angle θ_1_. The coated side is facing a sample tray containing the media whose refractive index is being measured. The reflected beam is collected by the receiving mirror RX. Reprinted from [17] with permission from AIP Publishing.

Another setup to consider, since it is the standard for SPR-based systems, is the Kretschmann configuration (Figure 5). This design involves the use of polarized light directed onto a coated prism at different incident angles, monitoring the reflectance power at the corresponding angle. In this case, the resonance will typically be observed as a dip at a given angle, and the SRI variations will be measured as a shift in the angle at which the said dip is measured. Alternatively, a broadband light source can be used in the same setup in order to monitor the spectra, such as in the setups explained previously.

In contrast to SPR, for which there are only a few coating materials considered appropriate for resonance generation (with gold being the standard), several materials have been successfully studied for LMR generation. Given the optical parameters required for a coating material to be able to support LMR generation, most of the research has been focused on metal oxides such as SnO_2_, TiO_2_, or ZnO; however, other materials such as graphene oxide or polymer-based films have also been tested, as will be described in the next section. The choice of the coating material will directly affect the performance and the validity of the sensor for the target application. Accordingly, a thorough study of the different materials used for this purpose is presented, indicating the main characteristics of each coating and some of the most relevant sensors developed with them.

## 3. Coating Materials

The following sections will describe the principal characteristics of each coating and their main achievements, as well as their differences. Some of the proposed applications and their performance as sensors will also be explained. Firstly, we present the studies that made use of several metal oxides coatings. Then, we discuss different works based on polymeric films. Finally, in the last section we group the works that are based on novel materials that could not be classified as an independent category.

### 3.1. Indium Tin Oxide (ITO)

**Figure 6:**
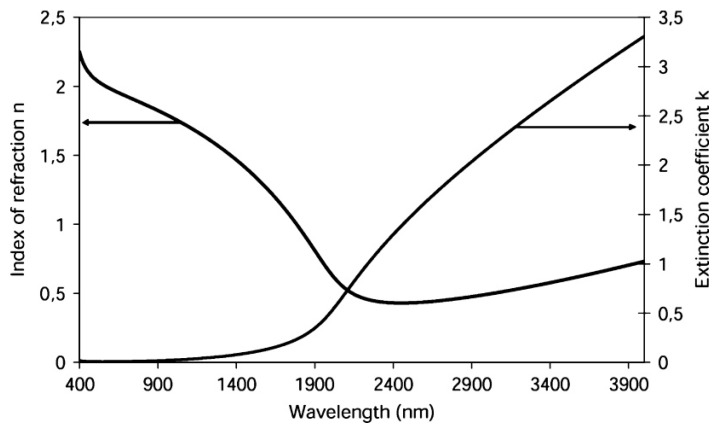
ITO refractive index curve. Shown are the optical properties suitable for the generation of LMR and SPR at shorter and longer wavelength ranges, respectively. Reprinted from [18] with permission from IEEE.

Indium tin oxide is a well-known transparent conductive oxide (TCO) that is widely used in optics and electronics. One very interesting characteristic of ITO is the fact that in can generate both LMR and SPR, as it fulfills the requisites for the generation of both resonance, with LMR being generated at lower wavelengths and SPR at wavelengths above 2000 nm [18] (see Figure 6). Over the last decade, several sensors using ITO as the coating material for the generation of LMR have been studied. ITO has been used on multimode fibers of different diameters [7,19,20], as well as D-shaped fibers [16,21] or glass slide in Kretschmann configuration [22]. Most authors have studied ITO as a coating material by fabricating a thin film by means of sputtering [16,23,24] or thermal evaporation technique [7], however other techniques such as dip coating have also been used [19]. While most studies have been focused on homogeneous ITO thin films, some works have been reported using nanoparticle-based ITO coatings [7].

The deposition of ITO on flat surfaces has been reported as a successful mechanism for LMR generation. Using a Kretschmann configuration [17], ITO thin film proved to be able to generate resonance in both TE and TM polarizations, obtaining sensitivities of 700 and 1200 nm/RIU, respectively, while a SPR is simultaneously observed with a sensitivity of 8300 nm/RIU for an SRI of 1.47. Recently, microscope coverslips coated with an ITO layer have been used as planar waveguides in a fiber setup to induce LMR [25], obtaining high sensitivity values (1405 and 920 nm/RIU for TE and TM modes, respectively). A similar setup using either a microscope glass slide or a coverslip coated with ITO and located in a poly(methyl methacrylate) (PMMA) block was used for the fabrication of a relative humidity sensor based on LMR [26].

One of the applications of ITO coatings for the fabrication of LMR-based sensors is the measuring of relative humidity (RH). In [19], a segment of cladding-removed 200/225 μm core/cladding MMF coated with ITO was used for the fabrication of a RH sensor with a sensitivity of 0.283 nm/%RH. This research also showed how the fabrication of an overlay of a material highly sensitive to humidity as PAH–PAA over the ITO coating improved the sensitivity to 0.833 nm/%RH. The same fiber configuration was also used for the fabrication of a turbine oil degradation sensor in [27]. Here, the reported device was capable of measuring the degradation of oil by measuring the LMR shift induced by the variation on the RI as a function of the oil degradation, obtaining a sensitivity of 0.15 × 10^−3^ nm/h (each hour of degradation induced a 0.15 × 10^−3^ nm shift in average).

ITO-coated MMF has also been used for other interesting applications, such as the detection of hydrogen (H_2_) [7]. This work compares the performance of three H_2_ sensors using a LMR support coating that consisted of an ITO thin film, ITO nanoparticles (NPs), and a layer of ITO NPs over an ITO thin film. These devices obtained sensitivity values of 0.32, 0.58, and 0.71 nm/ppm, respectively, proving that the use of ITO NPs enhances the sensitivity of the layer in the presence of H_2_.

Some novel setups have been designed for the improvement of the sensitivity of LMR-based sensors. In particular, in [14] the fabrication of an ITO coating on a tapered MMF end was proposed to observe LMR in a reflection setup. This configuration achieved a theoretical sensitivity of 12,005 nm/RIU, which is 1.4 times higher than the value simulated for a straight fiber.

**Figure 7:**
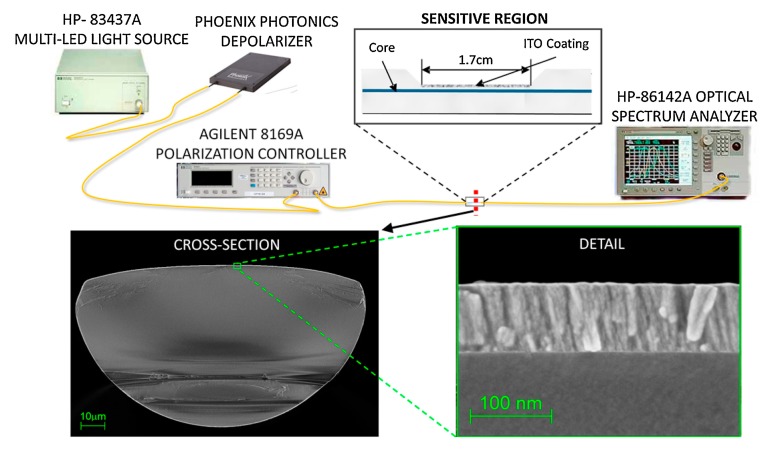
Example of LMR-based refractometer using a D-shaped fiber. A 17-mm long segment of side-polished single-mode fiber (SMF) was coated with an ITO thin film. The geometry of this structure can be appreciated in the cross-section image obtained by SEM. Reprinted from [16] with permission from Elsevier.

As mentioned before, one of the most relevant differences in practice in LMR compared to SPR is the fact that LMR can be observed in both TE and TM modes, making it easier to observe them without the use of polarized light. However, as the LMR can be generated at different wavelengths for each mode (TE and TM), the isolation of modes facilitates the possibility to obtain narrower resonances with higher sensitivity. This allows a better figure of merit (FOM) to be obtained, defined as the sensitivity/FWHM (full width at half maximum) ratio, as the ratio increases for more sensitive and narrower resonances. For this purpose, D-shaped fibers have been used for the fabrication of LMR-based sensors (Figure 7). In particular, a refractometer based on a D-shaped fiber narrowed the width of a LMR to 5.3 nm and reported a sensitivity of 8742 nm/RIU in the range of 1.36–1.38 RI [28]. A more recent work [16] reported a sensitivity of 136,276 nm/RIU in the range of 1.447–1.449 RI, with a peak of 304,361 nm/RIU. Here, it is important to consider the measuring SRI range, because a characteristic behavior of LMR is that the sensitivity increases at high RI ranges, which is confirmed by these results.

Several biosensors based on ITO thin films have also been reported in the literature. A C-reactive protein (CRP) sensor was designed using a combination of ITO and poly(allylamine hydrochloride–poly(sodium 4-styrenesulfonate) (PAH–PSS) coatings on a D-shaped fiber [21]. This device showed a sensitivity of up to 169.93 nm/mg L^−1^ and achieved a limit of detection (LOD) of 0.0625 mg/L. Another example is an immunoglobulin G sensor fabricated on an ITO-coated MMF, which was functionalized by adding a poly(methyl methacrylate) layer and immobilized antibodies, achieving a LOD of 3.5 μg/L [29].

From another perspective, some authors have taken advantage of the electrical conductivity of ITO for the development of LMR-based electrochemical sensors using ITO-coated MMFs [20,24,30,31]. For instance, an optical ketoprofen sensor was reported by Smietana et al. in [20]. This device monitored the electropolymerization of ketoprofen on an ITO coating showing, a LOD of 0.536 mM.

As a summary, the most relevant studies reported using ITO coatings for the fabrication of LMR-based sensors are shown in Table 1.

### 3.2. Tin Oxide

**Figure 8:**
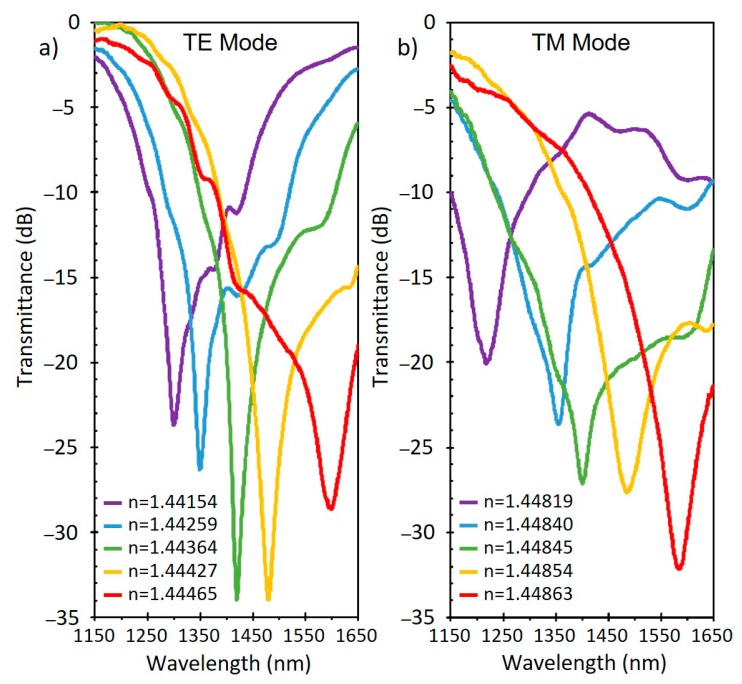
Transmittance of a D-shaped fiber with a tin oxide thin film with different SRI. Shown is the LMR corresponding to the TE mode (**a**) and TM mode (**b**). Reprinted from [32].

Another widely used material for the fabrication of LMR refractometers is tin oxide. It presents a number of similarities in comparison with ITO, since tin oxide is one of the components of ITO. Optically, SnO_2_ presents a slightly higher refractive index, which according to theory, permits higher sensitivities to SRI to be obtained [6]. This idea has been experimentally proven in several works using D-shaped fibers coated with a SnO_2_ thin film. In particular, a sensor working in the 1.321–1.326 RI (refractive index) range obtained a sensitivity of 14,501 nm/RIU [16], while a later work experimenting in the 1.441–1.449 RI range (Figure 8) reported a maximum sensitivity of 1,087,889 nm/RIU [32]. Such results imply that the refractive index could be measured with a resolution of 9.19 × 10^−10^ using an OSA with a standard resolution of 1 picometer.

A tin oxide thin-film-based LMR refractometer was also used to monitor turbine oil degradation [27]. In this case, the sensitivity was improved up to 0.27 × 10^−3^ nm/h (each hour of degradation induced a 0.27 × 10^−3^ nm shift on average. This material has also been reported for the fabrication of relative humidity sensors. In this sense, an etched SMF coated with a SnO_2_ thin film of 140 nm demonstrated a sensitivity of 1.9 nm/%RH [33].

In the field of biosensing, SnO_2_ has also been frequently studied. An immunoglobulin G (IgG) sensor using tin oxide over two different fiber structures was reported in [29]. Here, a configuration based on a 200 μm core MMF was capable to achieve a LOD of 0.9 μg/L, while the setup based on a D-shaped fiber reached a LOD of 0.15 μg/L, which are both comparatively better than the results obtained using an ITO coating. This improvement is a direct consequence of the higher sensitivity of tin oxide coating based refractometers. Additionally, a D-dimer sensor was studied in [34] using a tin-oxide-coated D-shaped fiber. This work presents LODs of 10 ng/mL and 100 ng/mL when tested in buffer and human serum, respectively, with response times of 10–20 min, which fit the range of other D-dimer detection platforms.

Most of the analyzed studies report SnO_2_ coatings fabricated by means of sputtering. However, there are other methods available for this purpose. Layer-by-layer (LbL) has also been proven as a valid option for the fabrication of SnO_2_ coatings. In [35], a 600 μm core MMF coated with SnO_2_–PSS bilayers was used in the design of a fiber refractometer in the range of 1.33–1.38 RI. This device reached a sensitivity of 4080 nm/RIU, but this value was increased up to 4704 nm/RIU when a SnO_2_ NP (nanoparticles) layer was added to the previous film.

Other dip coating techniques have also been described for the fabrication of LMR-based sensors using SnO_2_ nanoparticles. In [36], four possible combinations of a dual coating were tested for the development of an arsenite sensor. The two possibilities for the first layer were a tin oxide thin film or a SnO_2_ NP coating. Then, two different options were used for the second layer: one was a α-Fe_2_O_3_ NP layer, and the second was a core-shell nanostructure comprising α-Fe_2_O_3_ NP core with a SnO_2_ shell, abbreviated as α-Fe@Sn core–shell (CS). The combination of SnO_2_ NPs and α-Fe@Sn CS, which achieved the highest performance, allowed the generation of LMR at a wavelength of 370 nm. This device presented a sensitivity of 1.31 nm/μg L^−1^, with a LOD of 0.99 μg/L.

The above are not the only cases where two different layers were combined to improve the characteristics of the sensors. The use of a graphene oxide (GO) overlayer onto a 220-nm thick SnO_2_ thin film that proved to improve the sensitivity to ethanol by 210% is also described in [37].

As explained previously, one of the multiple reported configurations consisted of a planar waveguide (coverslip) where light was coupled from and to a MMF [25]. In this case, the coverslip was coated with SnO_2_, obtaining sensitivities of 800 and 1510 nm/RIU for the TE and TM modes, respectively. Another promising structure consisting of a multimode–coreless–multimode (MCM) fiber was also recently reported in [38]. In this configuration, where a 20 mm segment of SnO_2_-coated coreless fiber is fused to a couple of MMF pigtails, the proposed device reached a sensitivity of 7,346 nm/RIU when characterized as a refractometer. Additionally, the device was also used for the fabrication of a IgG detector, obtaining a LOD of 0.6 mg/L. A comparison of the performance of all the discussed sensors is shown in Table 1 at the end of the document.

### 3.3. Indium Oxide

**Figure 9:**
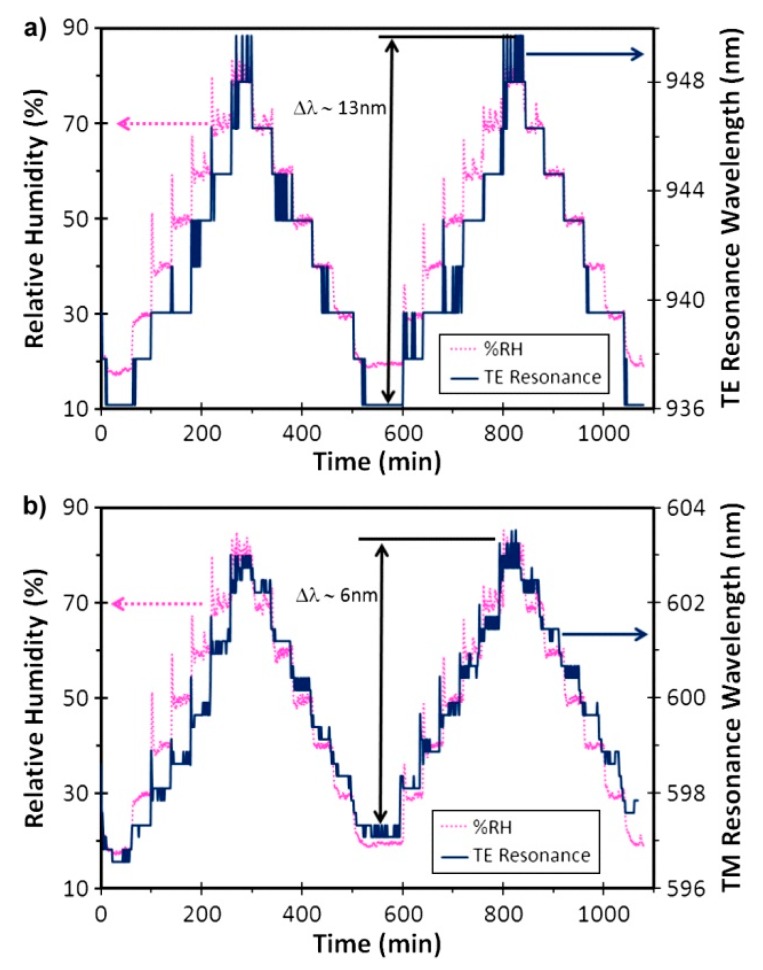
Relative humidity sensitivity test preformed for a In_2_O_3_-based sensor. The curve obtained from the LMR probe closely follows the one provided by the electronic RH sensor in both TE (**a**) and TM (**b**) modes. Reprinted from [39] with permission from IEEE.

The other component of ITO, indium oxide (In_2_O_3_), has not received as much attention as ITO or tin oxide in the LMR field. Most of the reported devices are associated with humidity or refractive index sensors (see Table 1). An early work demonstrated the fabrication of In_2_O_3_ coatings on a 200-μm core diameter CRMOF using the dip coating method [39]. In this case, three refractometers with increasing In_2_O_3_ film thickness were manufactured. All three devices presented 2 resonances, corresponding to the TE and TM modes, which could be observed separately at certain wavelengths. As the film thickness increased, the spectral positions of these resonances shifted to a longer wavelength. The sensor with the thickest coating (86 nm) presented resonances in the longest wavelengths (950 and 1400), which caused these resonances to have the highest sensibility (4255 and 4926 nm/RIU). This behavior is explained [6] by the fact that the LMR located at longer wavelengths show greater sensitivity than when they are observed at shorter wavelengths, due to having a thinner coating. This extensive work also demonstrated the response of this device to several VOCs (volatile organic compounds such as isopropyl alcohol, ethanol, methanol, and acetone), along with the cross-sensitivity to RH (Figure 9) and temperature. Figure 9 also shows the fast dynamical response of these sensors, which was faster than the 1 min resolution of the test.

A research work where ITO thin films were fabricated using the dip coating technique for the development of RH sensors also described the utilization of In_2_O_3_ for the same purpose [19]. In this case, an 85 nm layer of In_2_O_3_ was deposited onto a 200 μm CRMOF, resulting in two dips corresponding to the LMR caused by the two propagation modes, as explained above. A second device was designed by depositing a 115 nm layer of ITO via a similar procedure. These probes were later coated by PAH–PAA coatings by means of LbL to observe the effects of such overlays on the response of RH. Prior to the deposition of the polymeric coatings, the sensitivity to SRI variations was tested, showing that In_2_O_3_ reached higher values (4000 and 3333 nm/RIU for TE and TM modes, respectively) than ITO (1520 nm/RIU). In contrast, when the response to relative humidity was studied, it was seen that the resonance values generated by In_2_O_3_ (0.133 and 0.042 nm/%RH) showed lower sensitivity than the ITO probe (0.283 nm/%RH). This difference in the sensitivity response to SRI and RH is explained by the more porous nature of the ITO coating, which permits greater penetration and interaction with water molecules, inducing a greater LMR wavelength shift. However, when the RH-sensitive PAH–PAA coating was added, the response of the In_2_O_3_ sensor was better. In particular, the resonance located at a longer wavelength (corresponding to the TE mode) in the case of In_2_O_3_ reached a sensitivity of 0.935 nm/%RH, whereas the device based on the ITO and PAH–PAA layers obtained a sensitivity of 0.833 nm/%RH.

Recently, a new approach was taken regarding indium oxide coatings for LMR generation. In this case, the In_2_O_3_ films were fabricated by a sputtering system onto microscope glass slides and coverslips [40]. These devices acted as planar waveguides when light was launched laterally from an optical fiber on one side and collected back on the opposite side. The In_2_O_3_ film on the slides allowed the generation of LMR and the introduction of a light polarizer before the slide permitted the easy separation of propagation modes. A first probe was fabricated with a 276-nm thick In_2_O_3_ coating, which allowed the observation of a second-order LMR. This device obtained a sensitivity of 162 nm/RIU in the RI range of 1.33–1.357, which increased to 1375 nm/RIU when the RI range increased to 1.492–1.508. A second device was fabricated with a thinner coating (74 nm) in order to observe the first order resonance, which should be more sensitive. In this case, the sensitivity in the range of 1.33–1.357 reached a value of 929 nm/RIU, which is almost 6 times higher than the sensitivity obtained for the same RI range with the second-order LMR.

The discussed results obtained with In_2_O_3_-based coatings are summarized in Table 1 at the end of the text for a better comparison.

### 3.4. Zinc Oxide

The next relevant group of metal oxides used for the generation of LMR consists of those based on zinc oxide. This material also fulfills the optical requirements for the generation of LMR and can be fabricated by common methods such as sputtering or thermal evaporation. However, the first experimental demonstrations of LMR-based optical fiber refractometers using zinc oxide were reported only a few years ago [11,41]. These works relied on the capability of zinc oxide to react with hydrogen sulfide (H_2_S) gas for the design and fabrication of LMR-based hydrogen sulfide sensors. Different structures involving ZnO nanoparticles (NP) and ZnO nanorods (NR) were analyzed in these works in order to maximize the sensitivity of the LMR-based devices to H_2_S gas. A sensitivity of 1.06 nm/ppm was obtained when a ZnO NP layer was deposited directly on the fiber, which was increased up to 1.49 nm/ppm when a 12 nm thin film of ZnO was fabricated onto the fiber before the deposition of the NPs [41]. These figures were well above the results obtained when the same fiber structure was coated with silver thin film and an overlayer of zinc oxide to fabricate a SPR-based sensor, which could only achieve a sensitivity of 0.24 nm/ppm. Later, this mechanism was further enhanced by integrating ZnO nanorods, with a sensitivity enhancement of up to 4.14 nm/ppm [11]. A later work analyzed and compared the performance of these types of LMR-generating coatings as refractometers [8]. In this way, 400 μm core CRMOFs were coated with either a ZnO thin film, a ZnO thin film followed by a ZnO NP coating, or a ZnO thin film followed by a ZnO NR layer. All three structures were fabricated in a way that they presented LMR in a close wavelength (between 350 and 390 nm), so that their performance could be compared more directly. This study showed that a single zinc oxide thin film could provide a sensitivity of 760 nm/RIU, which could be increased to 950 nm/RIU by adding a layer of ZnO NPs and could be further improved using a layer of ZnO nanorods (1160 nm/RIU).

Aiming to improve the performance of LMR-based refractometers, ZnO thin film was also used in a novel structure [42]. This structure consisted of a CRMOF with a 400 μm core diameter, which was bent in order to achieve a U-shaped fiber, using a dual sensing approach to enhance the accuracy of the refractometer, measuring the LMR shift and the absorbance variation simultaneously. Additionally, this paper also introduces a novel approach to the use of LMR-based sensors, determining the SRI function of the variation of FWHM of the LMR instead of the wavelength shift. It is claimed that the use of FWHM provides 4 times higher sensitivity than the measured wavelength shift (895 nm/RIU compared to 220 nm/RIU, respectively).

Although zinc oxide coatings have not been as extensively reported for the fabrication of LMR-based sensors as the previous cases, they have already been considered for the development of LMR-based biosensors. A cortisol sensor using ZnO thin film and a molecularly imprinted polymer layer (MIP) was developed on a MMF [43]. In this work, it was demonstrated that a variation of the concentration of cortisol could induce a shift above 51 nm in a LMR, providing a sensitivity of up to 12.86 nm/log (g/mL) and a LOD of 25.9 fg/mL. The tests demonstrated the durability and stability of the device, which also showed a response time of 20 s. Similarly, a urinary p-cresol sensor was developed, which also relied on the MIP mechanism [44]. In this case, a zinc oxide and molybdenum sulfide (MoS_2_) nanocomposite was used for the fabrication of a LMR support coating. MoS_2_ was included in the nanocomposite for the purpose of altering the refractive index of the coating, improving the sensitivity of the device. In this way, the proposed sensor achieved a sensitivity of 11.86 nm/μM, with a LOD of 28 nM.

Zinc oxide thin film has also been considered for the development of LMR-based pressure sensors [45]. This theoretical work proposed the fabrication of a hafnium dioxide (HfO_2_) overlay on top of the zinc oxide thin film in order to maximize the sensitivity. With this structure, a sensitivity of 837 nm/MPa (and a maximum of 2000 nm/MPa) was calculated when a LMR-based refractometer was inserted in a rubber block, whose refractive index was dependent on the applied pressure. A summary of the LMR-based devices using ZnO thin films can be found in Table 1.

It is interesting to note that some coatings based on doped zinc oxide have also been studied for the development of LMR-based sensors. An aluminum-doped zinc oxide (AZO) thin film was proposed for the fabrication of LMR-based refractometers on a MMF tip. Theoretical simulations reported a sensitivity of 3500 nm/RIU, increasing to 8500 nm/RIU with the utilization of a dual coating (AZO–TiO_2_) [15]. Later, an experimental work showed the feasibility of the utilization of AZO for the generation of LMR-based refractometers by means of a 167 nm thin film [46]. This work showed the simultaneous generation of 3 LMR in the VIS-NIR range (visible and near-infrared spectrum), with a maximum sensitivity of 1153.6 nm/RIU for the lower-order resonance.

Recently, AZO coatings have been used in single–multimode-single mode (SMS) fiber setups [47]. First, the performance of two different multimode fibers was studied. A CRMOF (which had been etched for cladding removal) was used to fabricate a device that achieved a sensitivity of 937.8 nm/RIU. Substituting this fiber with a no-core fiber (NCF), the sensitivity increased to 1002.1 nm/RIU. This improvement was attributed to the larger diameter of the NCF (125 μm vs. 105 μm in the CRMOF), which also led to wider resonance. After optimizing the structure, the study compared the results when an AZO with a higher aluminum content was used for the fabrication of the coating. For this purpose, two targets with different Al concentrations (98:2 and 92:8) were used in the RF sputtering deposition process. It was shown that a higher aluminum content induced an improvement in the sensitivity of the refractometer, which reached 1214.7 nm/RIU.

Another well-known variant of zinc oxide, indium–gallium-doped zinc oxide (IGZO) has also been studied as a LMR support material [48]. IGZO coatings were fabricated on a 200 μm core diameter MMF and a series of D-shaped fibers with the purpose of fabricating LMR-based refractometers. On MMF, a LMR was observed at a wavelength of 640 nm. This device showed a sensitivity of 1666 nm/RIU when the fiber was immersed in solutions of RI varying between 1.33 and 1.45. Later, a series of D-shaped fibers was coated with IGZO to obtain LMR in the wavelength range between 1150 and 1650 nm. This configuration increased the sensitivity up to 12,929 nm/RIU. This paper also researched the impact of the polishing depth in the D-shaped fibers on the LMR, concluding that a deeper polishing depth induces a greater amplitude in the LMR and may alter its shape (generating “side-lobes”), but does not have a significant impact on the sensitivity of the LMR to SRI.

### 3.5. Titanium Dioxide

It was mentioned in the previous section that a structure consisting of a TiO_2_ film over an AZO layer was considered for the design of LMR-based refractometers. Titanium dioxide itself is also considered a very promising material for the development of optical LMR sensors, since it possesses a high refractive index and because TiO_2_-based coatings can be fabricated in a number of ways, such as by sputtering or layer-by-layer. An early work presented a TiO2/PSS thin film fabricated by LbL technique onto a 200 μm core diameter CRMOF. This device enables the generation of a LMR at a wavelength of 1100 nm, achieving a sensitivity of 2872 nm/RIU [49]. A recent work using a similar coating on a 600 μm core CRMOF obtained a sensitivity of 6754 nm/RIU at a wavelength of 650 nm [50]. In this case, a reflection setup was used for the characterization of the device, which also presented thinner coatings than the previous case (126 μm instead of 460 μm). These conditions permitted the observation of lower-order LMR, which show greater sensitivity. Another work presented the use of the reactive sputtering technique for the fabrication of a TiO_2_ thin film on a D-shaped fiber [51]. This 84 nm film generated a resonance at a wavelength of 1300 nm, but showed a sensitivity lower than the previous case of only 4122 nm/RIU. Following the experiences described with the previous materials, the use of a D-shaped fiber should not decrease the sensitivity of the sensor. The fact that a different deposition technique and different reagents are used in this research implies that the optical properties of the fabricated film may be quite different, which could have caused this unexpected result.

Another experimental use of TiO_2_ for the fabrication of optical LMR sensors was reported, seeking to design an ammonia sensor [52]. This work reports a TiO_2_ based coating deposited onto a tapered SMF by liquid phase deposition (LPD) process. This coating contains porphyrin, which is capable of interacting with ammonia as a functional material. This coating induces a LMR at 850 nm, which shifts in the presence of ammonia, obtaining a LOD of 0.116 ppm.

**Figure 10:**
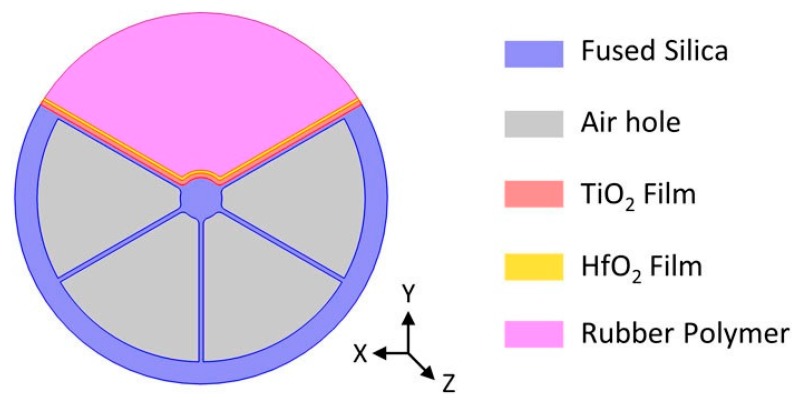
Cross-section of the grapefruit PCF structure proposed for the fabrication of a LMR-based pressure sensor. Reprinted from [54].

It should be noted that TiO_2_ thin films have also been used for the theoretical study of new fiber structures to fabricate LMR-based refractometers. The use of TiO_2_ coatings on a grapefruit PCF (photonic crystal fiber) with an exposed core has been recently reported [53]. This study suggests that appropriate waveguide selection could improve the sensitivity of LMR-based refractometers to 68,000 nm/RIU using a 100-nm thick TiO_2_ film. It also suggests the fabrication of a HfO_2_ layer on top of the TiO_2_ film, since the appropriate combination of thickness (80/20 nm) enhanced the sensitivity to 84,000 nm/RIU, with a maximum peak of 140,000 nm/RIU. A similar work proposed the addition of a rubber layer on top of the TiO_2_/HfO_2_ coating with a varying RI in the pressure application function (Figure 10) [54]. This would allow the fabrication of a pressure sensor that could achieve a sensitivity of 5 μm/MPa, according to the simulations.

Table 1 at the end of the text presents a brief summary of the devices presented in this section.

### 3.6. Polymers

**Figure 11:**
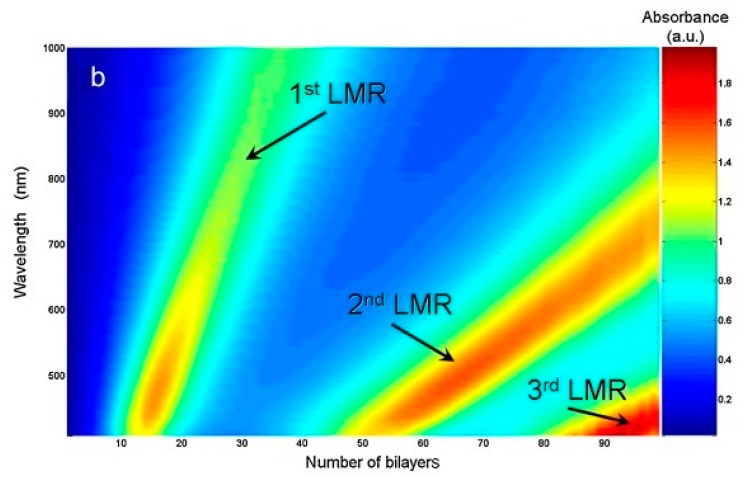
Transmittance spectra of a 200 μm CRMOF as polyallylamine hydrochloride (PAH) and polyacrylic acid (PAA) bilayers are being deposited on it. Several absorption bands can be observed, corresponding to LMR. Reprinted from [55] with permission from Elsevier.

Previous sections have dealt with coatings based on metal oxides for the generation of lossy mode resonance. It should be noted that some of the discussed coatings also included polymeric layers in their composition, which are mainly associated with sensing applications. In fact, LMR generation is not restricted to the use of metal oxides. Polymeric films are also capable of supporting LMR generation on their own, as long as their optical properties match the required conditions. For instance, a LMR-based sensor using polymeric coatings was reported for the sensing of pH [55]. A coating consisting of PAH–PAA bilayers deposited by LbL was chosen due to the swelling–deswelling behavior it presents with variation in pH. This change in the thickness of the coatings induces a wavelength shift in the LMR, and thus acts like a pH monitor. Figure 11 shows the transmission spectra on the fiber as PAH–PAA bilayers are added. It can be seen how several LMR are generated as the coating thickens, with each resonance showing a different slope. It is worth noting that the first LMR has the greatest slope, and the following ones present lower values. This slope indicates the sensitivity of the LMR to the thickness variation, is related to the sensitivity to SRI, and agrees with the premise that the lower order LMR are more sensitive. Following this procedure, two different probes were fabricated with 100 and 25 polymeric bilayers, respectively, in order to generate a LMR of second and first orders in the 500–700 nm range. These devices showed sensitivity values to pH 3-6 of 25 and 36.67 nm/pH, respectively.

A different kind of polymeric coating was also studied for the development of pH optical sensors, including gold nanoparticles in the polymeric matrix [56]. A PAH–PAA-Au NP coating deposited by layer-by-layer method on a CRMOF induced LMR at 750 nm and localized surface plasmon resonance (LSPR) at 530 nm. This probe was submerged in buffer solutions with pH values ranging between 4.0 and 6.0, showing a wavelength shift of both resonances as a function of the pH of the solutions. In particular, LSPR and LMR showed sensitivity values of 0.75 nm/pH and 67.35 nm/pH, respectively.

For the same purpose, D-shaped fibers were also coated with PAH–PAA bilayers. In this case, the pH of the solutions involved in the LbL fabrication process was adjusted in order to obtain two probes working in two different pH ranges [12]. The first probe was designed to operate in a pH range of 7–8, while the second probe was conceived for operation in the pH range of 4–5. In the first probe, the LMR corresponding to the TM and TE reached sensitivities of 30 and 34 nm/pH, respectively. In the second probe, the two resonances obtained sensitivities of 61 and 69 nm/pH, respectively.

Polymeric coatings were also used for the measurement of other parameters, such as relative humidity [9]. As mentioned previously, PAH–PAA layers present a high sensitivity to relative humidity. The indicated work studied several probes coated with PAH–PAA bilayers characterizing LMR of first and second orders at different wavelengths. The device with the best performance obtained a sensitivity of 0.51 nm/%RH. This value fell behind the previously reported sensitivity of 0.935 nm/%RH when the probe contained a In_2_O_3_ thin film beneath the PAH–PAA coating [19]. These results demonstrate the importance of using a LMR support coating that presents a high sensitivity, in this case in combination with a polymeric coating that reacts with the measured parameter.

Nanoparticles were also included in the polymeric coating matrices for the fabrication of relative humidity sensors. A PAH–PAA-Ag NP coating, for instance, was fabricated on a 200 μm core CRMOF in order to simultaneously induce a LMR and a LSPR, thanks to the addition of silver NPs [57]. In this device, the LMR achieved a sensitivity to RH of 0.943 nm/%RH, while the LSPR remained almost invariant (0.06 nm/%RH). A similar behavior was reported in another work, where dual LSPR and LMR phenomena were observed due to the presence of gold nanorods (GNR) [58]. A LbL coating of gold nanorods embedded in PSS (PAH/GNR@PSS) was capable of generating LSPR in two bands (LSPR-T at 530 nm and LSPR-L at 720 nm, related to the transverse and longitudinal plasmons, respectively) and LMR at 850 nm. As in the previous case, the LSPR were not very sensitive to RH variations (below 0.13 nm/%RH). In contrast, LMR achieved a sensitivity value of 11.2 nm/%RH.

Table 1 presents a brief summary of these sensors.

### 3.7. Other Materials

Previous sections have summarized the most widely used materials for the fabrication of LMR-based sensors. Several metal oxides and some polymers present the ideal properties in terms of electrical dispersion and thin film fabrication possibilities, making them good choices for this purpose. However, there are other materials that were not cited in the previous sections that have gained more attention for the support of LMR in recent works. For instance, graphene oxide (GO), which has already been described above because of its use as an overlay to enhance the sensitivity of a SnO_2_ thin film based sensor, was also experimentally tested in [59]. Two different sensors were fabricated in this work using 8 and 20 bilayers of GO and polyethylenimine (PEI) by means of the LbL technique on a 200 μm core CRMOF. The number of bilayers was chosen to obtain LMR of different orders but at the same wavelength (550 nm) in both probes. The probes were later tested on solutions of increasing refractive indices, obtaining sensitivity values of 2631 and 12,460 nm/RIU for the devices with 20 and 8 bilayers, respectively.

Another interesting material that has been recently considered for the fabrication of LMR refractometers is copper oxide [25]. The high refractive index of this material reveals a great potential for the development of highly sensitive refractometers that are comparable to tin oxide.

**Figure 12:**
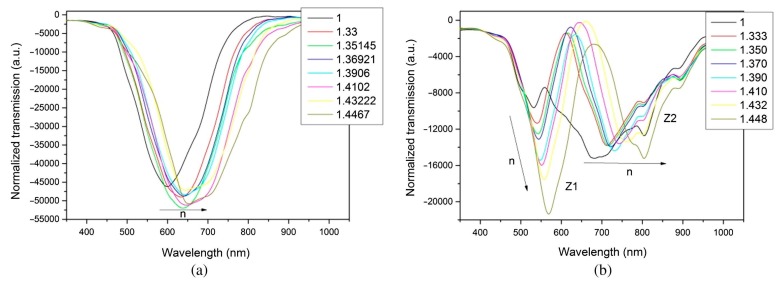
Transmission spectra of HfO_2_ (**a**) and a ZrO_2_ (**b**)-coated CRMOF for different SRI. Reprinted from [10] with permission from Elsevier.

A recent work [10] presented a number of coating materials that can be deposited by atomic layer deposition (ALD) and demonstrated their capability to fabricate LMR-based sensors. This deposition technique is chosen because of its capacity to control the growth rate with great precision, which is a key aspect for the fabrication of LMR-based sensors. ALD was used to fabricate hafnium oxide (HfO_2_) and zirconium oxide (ZrO_2_) thin films on a 400 μm core CRMOF. Figure 12 shows the transmission spectra for such devices when immersed on solutions of increasing SRI. Thin films of silicon nitride (Si_x_N_y_) were also fabricated by plasma-enhanced chemical vapor deposition (PECVP). This technique is not as precise as ALD, but in contrast it allows the fabrication of thicker coatings in a reasonable time. A fourth kind of probe consisted of the fabrication of a tantalum oxide (Ta_2_O_5_) film on top of the silicon nitride layer by ALD. This structure is designed so that a thick coating can be fabricated in a reasonable timeframe (Si_x_N_y_ by PECVD), while at the same time allowing fine-tuning with the addition of the Ta_2_O_5_ overlay with ALD. The ZrO_2_ probe obtained sensitivity values of 195 and 880 nm/RIU for the RI ranges of 1.33–1.45 and 1.41–1.43, respectively. In the case of the silicon-nitride-based sensor, it obtained sensitivity values of 289.5 and 593.5 nm/RIU for the same RI ranges. When the tantalum oxide film was deposited on the silicon nitride layer, the sensitivity of the device for the lower RI range increased to 1077 nm/RIU, but the opposite behavior was obtained for the 1.43–1.45 range as the sensitivity slightly decreased to 483 nm/RIU. Table 1 summarizes these results.

## 4. Conclusions

A number of materials have already been studied for the generation of LMR-based sensors. Each coating material has led to the development of LMR-based sensors for different purposes with diverse results. The increasing number of studies in this field during the last decade highlights the versatility of LMR for the development of sensors. In this sense, materials such as ITO, tin oxide, indium oxide, PAH, PAA, and even silver nanoparticles or gold nanorods have been used for the fabrication of relative humidity sensors [9,19,33,57,58].

Nanoparticles (NPs) have been widely used in layers for the fabrication of gas sensors, as this structure increases the interaction surface. For example, it was already mentioned that a combination of layers of SnO_2_ NPs and α-Fe@SnO CS facilitated the fabrication of an arsenite sensor [36]. It was also described how the addition of NPs to an ITO layer increased the performance of a hydrogen sensor [7]. The fabrication of a second layer of ZnO NPs and NRs on top of a zinc oxide thin film also increased the sensitivity of a hydrogen sulfide sensor [11,41].

Zinc oxide thin films have also been used in the fabrication of LMR-based sensors using molecularly imprinted polymers (MIPs) [43,44].

It should be noted that the described sensors have been fabricated using different deposition techniques. Those relying on dip coating or layer-by-layer techniques (such as the polymeric coatings) can be implemented faster than those relying on techniques such as sputtering deposition or ALD, for instance, which require a more economically demanding infrastructure.

The sensitivity of the lossy mode resonance to external refractive index variations has been extensively studied, especially in the range close to water (1.33), since most biological applications work in this range. For instance, a graphene oxide coating [59] provided a sensitivity of 2631 nm/RIU at a wavelength of 550 nm, while a TiO_2_/PSS film [50] produced LMR with a sensitivity of 6754 nm/RIU at 650 nm. Additionally, a In_2_O_3_-based refractometer [39] gave a value of 3003 nm/RIU at 600 nm, which increased to 4926 nm/RIU when a thicker coating shifted the LMR to 1400 nm; all of these examples used CRMOF. Using a D-shaped fiber setup, a refractometer based on an ITO thin film [28] gave a sensitivity of 8742 nm/RIU. That figure was almost doubled (14,501 nm/RIU) when a coating of tin oxide was used instead [16].

In terms of sensitivity, the highest figures were achieved with SnO_2_ coatings in both the refractive index range of water [16] and in the range of fused silica [32]. These results were due to the optical properties of the thin film, which were in agreement with the established theory that predicts that a LMR support coating with a high refractive index will lead to devices with high sensitivity to SRI variations [6]. Accordingly, several biosensors have been designed using tin oxide coatings as LMR support coatings, aiming to obtain the lowest possible limit of detection (LOD), as is the case D-dimer [34] and immunoglobulin G [29] sensors.

However, tin oxide optical properties are not as exceptional. In fact, there are other materials whose refractive indices do not differ much from that of SnO_2_ that are potentially capable of generating LMR with as good or even higher sensitivity. Materials such a copper oxide, which possesses a higher refractive index and a low extinction coefficient, could surpass the performance of tin oxide in the fabrication of lossy mode resonance based sensors. However, a potentially high sensitivity is not the only parameter to consider when it comes to finding the best material for the fabrication of sensors. Other considerations such as the porosity of the films, stability, fabrication process, and affinity with the functionalizing layer must be taken into consideration for the selection of the LMR support material. The possibility of designing a structure of two (or more) layers [10,37] is a promising approach for the optimization of these sensors. The phenomenon of LMR for the fabrication of optical sensors has already proved to have great potential, and the vast number of possible materials that can be used to design such sensors represents a limitless field of research. The future research on coating materials for LMR-based refractometers should probably be focused on finding materials with an optimal refractive index to maximize sensitivity, as well as on the study of more advanced structures, such as multiple layers.

## Figures and Tables

**Table 1:** Performance of the different sensors based on the LMR detailed in this paper. A wide range of applications has been developed based on this phenomenon using a variety of materials. The sensors are sorted by coating material, configuration setup, and application.

Material	Configuration	Refractive Index Range	Wavelength	Application/Sensitivity	Ref.
**ITO + PAH–PAA**	200 μm MMF	N/A	1430	Relative Humidity: 0.833 nm/%RH	[19]
**ITO**	200 μm MMF	N/A	750	Turbine oil degradation: 0.15 × 10^−3^ nm/h	[27]
**ITO**	200 μm MMF	N/A	550	Immunoglobulin G LOD: 3.5 μg/L	[29]
**ITO**	400 μm MMF	N/A	600	Ketoprofen: 1400.86 nm/M LOD: 0.536 mM	[20]
**ITO + ITO NPs**	600 μm MMF	N/A	400	H_2_ Gas: 0.71 nm/ppm	[7]
**ITO**	D-shaped	1.365–1.38	1250	Refractive Index: 8742 nm/RIU	[28]
**ITO**	D-shaped	1.447–1.449	1280	Refractive Index: 304,361 nm/RIU	[16]
**ITO + PAH–PSS**	D-shaped	N/A	1445	CRP: 10.61–169.93 nm/mg L^−1^ LOD: 0.0625 mg/L	[21]
**ITO**	Coverslip	1.33–1.37	800	Refractive Index: 1405 nm/RIU	[25]
**ITO**	Coverslip	N/A	570	Relative Humidity: 0.116 nm/%RH	[26]
**SnO_2_**	200 μm MMF	N/A	1100	Turbine oil degradation: 0.27 × 10^−3^ nm/h	[27]
**SnO_2_ + GO**	200 μm MMF	N/A	537	Ethanol: 0.525 nm/%Eth(v/v)	[37]
**SnO_2_–PSS + SnO_2_ NPs**	600 μm MMF	1.33–1.38	840	Refractive Index: 4704 nm/RIU	[35]
**SnO_2_**	Etched SMF	N/A	1550	Relative Humidity: 1.9 nm/%RH	[33]
**SnO_2_ NPs + α-Fe@Sn CS**	Unclad Fiber	N/A	370	Arsenite: 1.31 nm/μg L^−1^ LOD: 0.99 μg/L	[36]
**SnO_2_**	D-shaped	1.321–1.326	1380	Refractive Index: 14,501 nm/RIU	[16]
**SnO_2_**	D-shaped	1.448–1.449	1200	Refractive Index: 1,087,889 nm/RIU	[32]
**SnO_2_**	D-shaped	N/A	1445	Immunoglobulin G LOD: 0.15 ng/L	[29]
**SnO_2_**	D-shaped	N/A	1400	Dimer-D LOD: 10 and 100 ng/mL in buffer and human serum resp.	[34]
**SnO_2_**	MCM	N/A	750	Immunoglobulin G LOD: 0.6 mg/L	[38]
**SnO_2_**	Coverslip	1.33–1.37	900	Refractive Index: 1800 nm/RIU	[25]
**In_2_O_3_**	200 μm MMF	1.32–1.37	1400	Refractive Index: 4926 nm/RIU	[39]
**In_2_O_3_ + PAH–PAA**	200 μm MMF	N/A	1020	Relative Humidity: 0.935 nm/%RH	[19]
**In_2_O_3_**	Coverslip	1.333–1.357	730	Refractive Index: 929 nm/RIU	[40]
**ZnO**	400 μm U-shaped MMF	1.33–1.42	400	Refractive Index: 220 nm/RIU	[42]
**ZnO + ZnO NRs**	400 μm MMF	1.33–1.44	390	Refractive Index: 1160 nm/RIU	[8]
**ZnO–MoS_2_**	600 μm MMF	N/A	380	Urinary p-cresol: 11.86 nm/μM LOD: 28 nM	[44]
**ZnO + ZnO–PPY**	600 μm MMF	N/A	400	Cortisol: 12.86 nm/log (g/mL) LOD: 25.9 fg/mL	[43]
**ZnO + ZnO nanorods**	600 μm MMF	N/A	350	Sulfide gas: 4.14 nm/ppm	[11]
**ZnO + ZnO NPs**	600 μm MMF	N/A	370	Sulfide gas:1.49 nm/ppm	[41]
**AZO**	200 μm MMF	1.3334–1.4471	1200	Refractive Index: 1153.6 nm/RIU	[46]
**AZO**	SMS	1.365–1.405	1530	Refractive Index: 1214.7 nm/RIU	[47]
**IGZO**	D-shaped	1.39–1.42	1150	Refractive Index: 12,929 nm/RIU	[48]
**TiO_2_–PSS**	200 μm MMF	1.32–1.43	1100	Refractive Index: 2872.73 nm/RIU	[49]
**TiO_2_–PSS**	600 μm MMF	1.33–1.38	650	Refractive Index: 6754 nm/RIU	[50]
**TiO_2_**	D-shaped	1.333–1.398	1300	Refractive Index: 4122 nm/RIU	[51]
**TMPyP–TiO_2_**	Tapered SMF	N/A	850	Ammonia LOD: 0.16 ppm	[52]
**PAH–PAA**	200 μm MMF	N/A	610	pH (3–6): 36.67 nm/pH	[55]
**PAH–PAA**	D-shaped	N/A	1397	pH (4–5): 69 nm/pH	[12]
**PAH–PAA–AuNP**	200 μm MMF	N/A	750	pH (4–6): 67.35 nm/pH	[56]
**PAH–PAA**	200 μm MMF	N/A	700	Relative Humidity: 0.51 nm/%RH	[9]
**PAH–GNR@PSS**	200 μm MMF	N/A	850	Relative Humidity: 11.2 nm/%RH	[58]
**PAH–PAA–Ag NPs**	200 μm MMF	N/A	750	Relative Humidity: 0.943 nm/%RH	[57]
**Graphene Oxide (GO)**	200 μm MMF	1.39–1.42	550	Refractive Index: 12,460 nm/RIU	[59]
**CuO**	Coverslip	1.33–1.37	750	Refractive Index: 1537 nm/RIU	[25]
**ZrO_2_**	400 μm MMF	1.41–1.43	700	Refractive Index: 880 nm/RIU	[10]
**Si_x_N_y_ + Ta_2_O_5_**	400 μm MMF	1.33–1.35	770	Refractive Index: 1077 nm/RIU	[10]
